# Impulsivity Is Related to Prescription Opioid Misuse in People With Chronic Pain Through Pain Catastrophizing and Emotional Distress

**DOI:** 10.1155/prm/6315721

**Published:** 2025-09-23

**Authors:** R. Esteve, C. Ramírez-Maestre, E. R. Serrano-Ibáñez, A. E. López-Martínez

**Affiliations:** ^1^Facultad de Psicología y Logopedia, Andalucía Tech, Universidad de Málaga, Málaga, Spain; ^2^Instituto de Investigación Biomédica de Málaga y Plataforma en Nanomedicina (IBIMA Plataforma BIONAND), Málaga, Spain

**Keywords:** anxiety, depression, impulsivity, pain catastrophizing, prescription opioid misuse

## Abstract

**Background:** This study investigated the role of impulsivity as a personality variable predisposing to prescription opioid misuse. Pain catastrophizing, as well as anxiety, depression, and pain intensity, were postulated as potential serial mediators in this relationship.

**Methods:** The sample comprised 366 individuals with chronic pain. We conducted correlational and serial mediation analyses to investigate the relationships between the study variables.

**Results:** The results showed that pain catastrophizing partially mediated the relationship between impulsivity and prescription opioid misuse. Depression and anxiety also partially mediated the relationship between pain catastrophizing and prescription opioid misuse, although pain intensity did not mediate this relationship. Impulsivity was also indirectly associated with prescription opioid misuse through pain catastrophizing, anxiety, and depression.

**Conclusions:** Our findings highlight the key role of impulsivity in prescription opioid misuse and contribute to understanding its mechanisms of action. Based on these results, clinical interventions could target emotion-related impulsivity and cognitive control deficits to reduce rumination. Future research could investigate the relationships identified in this study using the specific dimensions of impulsivity.

## 1. Introduction

Chronic pain is a worldwide health problem [1]. The pharmacological management of chronic pain often includes the prescription of opioids [2]. However, there is ample evidence that people with chronic pain frequently misuse prescription opioids and that misuse has adverse consequences on their well-being [3]. In this study, we adopt the definition of opioid misuse proposed by Vowles et al. [4], which refers to any use that deviates from the prescribed pattern, regardless of the presence or absence of harmful effects. Thus, the variables associated with prescription opioid misuse should be determined to prevent its appearance and aggravation [5]. There is strong evidence that psychological variables play a crucial role in the development and aggravation of prescription opioid misuse (e.g., [6]).

Impulsivity is a dispositional variable consistently associated with addiction and substance abuse in general [7] and prescription opioid misuse in particular [8]. Impulsivity has been defined as a predisposition toward rapid, unplanned reactions to internal or external stimuli without regard to the negative consequences of these reactions to the individuals themselves or to others [9]. The few studies on the relationship between prescription opioid misuse and impulsivity have shown that only a reduced set of dimensions are relevant. Marino et al. [10] found that of the three subscales of the Barratt Impulsiveness Scale (BIS) for adults (i.e., attentional impulsivity, motor impulsivity, and nonplanning impulsivity) [11], only Attentional Impulsivity—the capacity to control attention—was significantly associated with the risk of opioid misuse in patients with chronic pain. Vest et al. [8] found that, of the four dimensions of impulsivity measured by the UPPS Impulsive Behavior Scale [12], (i.e., urgency, lack of premeditation, lack of perseverance, and sensation seeking), only urgency—defined as the tendency to react impulsively in response to negative emotional states—was associated with the risk of future and current misuse of prescription opioids, as well as symptoms of current opioid use disorder. Sensation seeking was also associated with current misuse.

Pain catastrophizing is an exaggerated negative mental set triggered during actual or anticipated painful experiences [13] and comprises three elements: rumination, magnification, and helplessness [13]. Numerous studies have shown that pain catastrophizing is consistently related to increased emotional distress (anxiety and depression) and pain intensity (for a review, see [14]). Petrini and Arendt-Nielsen [15] recently conceptualized pain catastrophizing as unproductive problem-solving or deficient emotion regulation leading to distress and heightened pain perception. Catastrophic thinking about pain is linked to the misuse of prescription opioids in individuals with chronic pain (e.g., [16]) and could lead to prescription opioid misuse through heightened levels of pain intensity, pain sensitivity, and negative affect [17]. In this line, Arteta et al. [18] showed that anxiety and depression were both mediators of the relationship between pain catastrophizing and opioid misuse. However, in a sample of people with persistent pain referred for treatment for opioid misuse, Baranoff et al. [19] found that depression contributed to misuse over and above anxiety and pain catastrophizing. Regarding the relationship between negative affect and opioid misuse, several theoretical frameworks have highlighted the role of negative reinforcement in maintaining substance misuse and addiction. These frameworks suggest that individuals learn to associate opioid use with the alleviation of aversive internal experiences, such as negative affect and pain (e.g., [20, 21]).

Several authors have suggested that future research could investigate how personality traits (e.g., impulsivity) that are associated with an increased risk of opioid misuse may predispose to it through increased pain catastrophizing [17, 18]. Impulsivity and pain catastrophizing may be linked through their attentional components. Deficits in attentional control are a major component of impulsivity [22], and emotion-related impulsivity is conceptually and empirically associated with rumination since both involve failures of top-down control over responses to emotion [23]. Pain catastrophizing is characterized by the inability to divert attention away from pain-related thoughts and pain (i.e., rumination) [13] and by difficulty in disengaging from pain cues [24]. Pain catastrophizing and prescription opioid misuse may also be related through negative affect and pain intensity [17, 18].

Thus, the main objective of the present study was to determine whether impulsivity influences opioid misuse through pain catastrophizing, emotional distress (anxiety and depression), and pain intensity. In line with prior research, the study hypotheses were as follows: pain catastrophizing would mediate the relationship between impulsivity and prescription opioid misuse (H1); and anxiety, depression, and pain intensity would mediate the relationship between pain catastrophizing and prescription opioid misuse (H2). As far as we know, no research exists on the mechanisms mediating the relationship between impulsivity as a personality differential variable and prescription opioid misuse.

## 2. Methods

### 2.1. Participants

A consecutive sample of 366 individuals with chronic noncancer pain participated in this study. Individuals were eligible for inclusion if they fulfilled the following criteria: experiencing chronic pain and under treatment with opioids for at least 90 days [25]; not diagnosed or treated for a malignancy, terminal illness, or psychiatric disorder; and able to understand the Spanish language. The doctors collaborating in the study invited 727 patients with chronic noncancer pain to participate. The research team contacted them and again screened the patients for eligibility. Of the initial pool of 727 individuals, 27 did not meet the inclusion criteria and were thus excluded from the study. When contacted, 334 individuals declined participation: 167 expressly refused, 79 did not attend the assessment session, 68 indicated they could not travel to the hospital to attend the evaluation session, and 20 did not reply to the phone calls. As a result, the final sample comprised 366 participants.

### 2.2. Questionnaires

#### 2.2.1. Demographic and Clinical Variables

Participants were interviewed to collect information on demographic and clinical variables, such as pain duration and medications. We determined the daily opioid dosage; converting it to oral morphine milligram equivalents (MME) using the recommended conversion factors [26].

#### 2.2.2. Impulsivity

We applied the Spanish version of the BIS for adults (BIS-11) [11, 27]. The BIS-11 comprises 30 items answered on a 4-point scale. The BIS-11 has three subscales [11]: nonplanning, motor, and attentional. It also provides a total score on impulsivity. In the current sample, the values of Cronbach's alpha were as follows: total scale, 0.80; nonplanning subscale, 0.48; motor subscale, 0.70; and attentional subscale, 0.51.

#### 2.2.3. Pain Catastrophizing

We administered the Spanish version of the Pain Catastrophizing Scale [13, 28]. It consists of three subscales assessing rumination, magnification, and helplessness, and also provides a total score on catastrophizing. The total score alone was used in this study. This scale is a 13-item measure in which respondents indicates on a 5-point scale the degree to which they experience various thoughts and feelings while in pain. In the current sample, the value of Cronbach's alpha was 0.92.

#### 2.2.4. Anxiety and Depression

We applied the Spanish version of the Hospital Anxiety and Depression Scale (HADS) [29, 30]. The HADS consists of two 7-item self-report scales, one assessing anxiety and the other assessing depression. Participants respond using a 4-point Likert scale. In this sample, Cronbach's alpha values indicated high internal consistency (*α* = 0.84, anxiety scale; *α* = 0.85, depression scale).

#### 2.2.5. Pain Intensity

Participants rated their mildest, average, and worst pain over the past 2 weeks, along with their current pain, using an 11-point Likert scale. We calculated these scores' average and obtained a composite pain intensity score [31]. In this sample, Cronbach's alpha value was 0.84.

#### 2.2.6. Prescription Opioid Misuse

We applied the Spanish version of the Current Opioid Misuse Measure (COMM) [32, 33]. The COMM consists of 17 items rated on a 0–4 scale. In the current sample, the value of Cronbach's alpha was 0.78.

### 2.3. Procedure

All the procedures followed the Declaration of Helsinki 1964 and its later amendments. This study is part of a larger project [34] that received ethical approval from both the Institutional Ethics Review Board of the University of Malaga (Reference: CEUMA 66-2019-H) and the Research Ethics Committee of the Province of Málaga (CEIP-281021). Participants provided signed informed consent before data collection, and we assured them that the information collected would remain confidential. The researchers met with the participating doctors, explained the eligibility criteria, and agreed on the procedures. At the end of their visit, the doctors explained the study's aims and invited eligible patients to participate. These patients provided their telephone numbers to schedule an appointment on another day. Demographic and clinical data were collected through an interview with a psychologist who also administered the instruments described in the following section. The recruitment process was conducted in three pain units from May 2021 to June 2023.

### 2.4. Data Analyses

Analyses were performed using the Statistical Package for Social Sciences (SPSS; Windows version 22.0, SPSS Inc., Chicago, IL). We computed descriptive statistics for the demographic, clinical, and other study variables. Furthermore, we calculated bivariate correlations among the continuous variables using Pearson's coefficient. Correlations were interpreted according to the guidelines proposed by Cohen [35], with values between 0.10 and 0.29 considered low, 0.30 to 0.49 considered moderate, and 0.50 to 1 considered high.

The study hypotheses were tested by performing four serial mediation regression analyses using Model 6 of the PROCESS 3.5. macro for SPSS [36]. This analysis examined relationships between one independent variable (*X*: impulsivity), one dependent variable (*Y*: prescription opioid misuse), and two simultaneous serial mediator variables (MV1: pain catastrophizing; MV2: in each analysis, depression, anxiety, or pain intensity). Serial mediation models assume that mediators directly affect each other and that the independent variable (impulsivity) influences mediators in a serial mode, which subsequently influences the dependent variable (prescription opioid misuse). We used bootstrap resampling techniques (with 10,000 resamples) with a 95% bias-corrected confidence interval (CI) to assess indirect effects [37]. Indirect effects were regarded as significant if the CIs did not include zero. To avoid measurement-associated problems, we mean-centered all the variables included in the mediation analyses.

We also used the Sobel test to assess the significance of partial mediation effects. The level of statistical significance was set at *p*=0.05. This procedure operates independently of the assumptions typically required for regression analysis. A priori power analysis was not conducted; however, the final sample size provided sufficient power for detecting indirect effects in serial mediation models, as estimated using a power analysis method based on Monte Carlo CIs performed with an application [38] developed by Schoemann et al. [39]. The results section provides the statistical power associated with each analysis.

## 3. Results

### 3.1. Descriptive and Correlation Analyses

Table 1 shows the participants' demographic and clinical characteristics.  Table 2 shows the means, standard deviations, and correlation analyses of the continuous variables.

The following statistically significant correlations were found: between impulsivity and pain catastrophizing and pain intensity (low); between impulsivity and anxiety, depression, and opioid misuse (moderate); between pain catastrophizing and depression, pain intensity, and opioid misuse (moderate) and anxiety (high); between anxiety and pain intensity and opioid misuse (moderate) and depression (high); between depression and pain intensity (low) and opioid misuse (moderate); and between pain intensity and opioid misuse (low).

Regarding hypothetical mediating variables, positive significant correlations were found between pain catastrophizing and anxiety (high), depression (moderate), and pain intensity (moderate). These correlations remained significant after controlling for impulsivity (with anxiety, pr = 0.50, *p* < 0.001; with depression, pr = 0.44, *p* < 0.001; with pain intensity, pr = 0.29, *p* < 0.001). These results indicate that one variable influences the other, supporting the use of serial multiple mediation analysis [36].

### 3.2. Mediation Analyses

We performed three serial multiple mediation analyses to test the study hypotheses. The study included two serial mediators: catastrophizing (*M*_1_), which was included in the three analyses, and depression, anxiety, and pain intensity (*M*_2_), which were included consecutively in the analyses.

**Figure 1:**
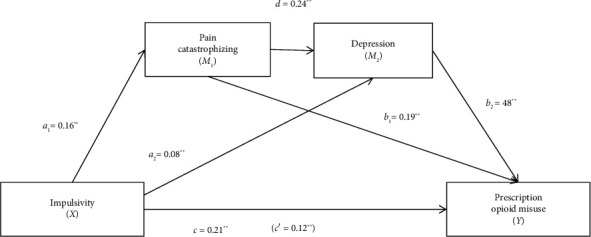
Diagram of the serial mediation model for pain catastrophizing and depression as mediators between impulsivity and prescription opioid misuse. ⁣^∗^*p* < 0.05; ⁣^∗∗^*p* < 0.001.

The total effect of the model with pain catastrophizing and depression as mediators was significant (*R*^2^ = 0.32, *F* [3, 362] = 55.50, *p* < 0.001). Pain catastrophizing and depression were significantly associated with prescription opioid misuse, and the direct effect (path c') of impulsivity on opioid misuse was significant, suggesting that pain catastrophizing and depression partially mediated the effect of impulsivity on prescription opioid misuse (Figure 1). The total indirect effect and all the specific indirect effects were statistically significant, as the 95% CIs of the point estimate did not cross zero (Table 3). There was a significant indirect pathway from impulsivity to prescription opioid misuse through pain catastrophizing, a significant indirect pathway from impulsivity to prescription opioid misuse through depression, and a significant indirect serial pathway from impulsivity to prescription opioid misuse through pain catastrophizing and depression. This analysis had high power (*a*_1_*b*_1_ = 0.99; *a*_2_*b*_2_ = 1.00; *a*_1_*db*_2_ = 1.00) to detect mediation effects at a significance level of 0.05.

**Figure 2:**
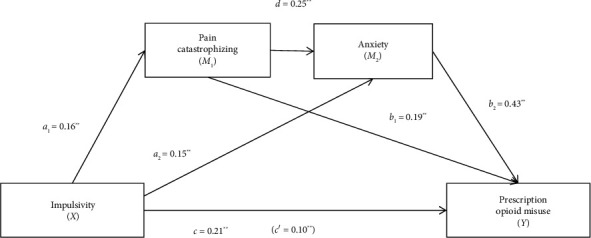
Diagram of the serial mediation model for pain catastrophizing and anxiety as mediators between impulsivity and prescription opioid misuse. ⁣^∗^*p* < 0.05; ⁣^∗∗^*p* < 0.001.

The total effect of the model with pain catastrophizing and anxiety as mediators was significant (*R*^2^ = 0.29, *F* [3, 362] = 49.78, *p* < 0.001). A significant association was found between pain catastrophizing and anxiety with prescription opioid misuse; the direct effect (path c′) of impulsivity on opioid misuse was significant, suggesting that pain catastrophizing and anxiety partially mediated the effect of impulsivity on prescription opioid misuse (Figure 2, Table 3). The total indirect effect and all the specific indirect effects were statistically significant, as the 95% CIs of the point estimate did not cross zero (Table 3). There was a significant indirect pathway from impulsivity to prescription opioid misuse through pain catastrophizing, a significant indirect pathway from impulsivity to prescription opioid misuse through anxiety, and a significant indirect serial pathway from impulsivity to prescription opioid misuse through pain catastrophizing and anxiety. This analysis had high power (*a*_1_*b*_1_ = 0.98; *a*_2_*b*_2_ = 1.00; *a*_1_*db*_2_ = 0.99) to detect mediation effects at a 0.05 significance level.

**Figure 3:**
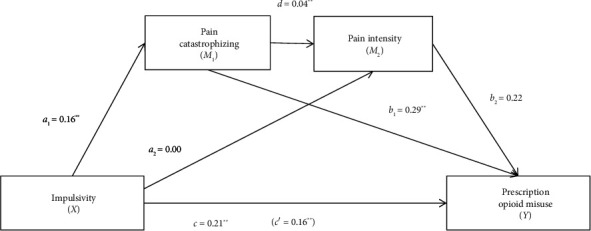
Diagram of the serial mediation model for pain catastrophizing and pain intensity as mediators between impulsivity and prescription opioid misuse. ⁣^∗^*p* < 0.05; ⁣^∗∗^*p* < 0.001.

The total effect of the model with pain catastrophizing and pain intensity as mediators was significant (*R*^2^ = 0.25, *F* [3, 362] = 39.22, *p* < 0.001). A significant association was found between pain catastrophizing and prescription opioid misuse; the direct effect (path c′) of impulsivity on opioid misuse was significant, suggesting that pain catastrophizing partially mediated the effect of impulsivity on prescription opioid misuse (Figure 3, Table 3). No significant associations were found between impulsivity and pain intensity or between pain intensity and prescription opioid misuse. The total indirect effect was statistically significant, as the 95% CIs of the point estimate did not cross zero (Table 3). The only significant indirect serial pathway was from impulsivity to prescription opioid misuse through pain catastrophizing. There was no significant indirect effect between impulsivity and prescription opioid misuse through pain intensity, or between impulsivity and prescription opioid misuse through pain catastrophizing and pain intensity. The power of this analysis to detect mediation effects at a significance level of 0.05 was as follows: *a*_1_*b*_1_ = 1.00; *a*_2_*b*_2_ = 0.01; *a*_1_*db*_2_ = 0.12.

## 4. Discussion

This study investigated the role of impulsivity as a dispositional variable which could contribute to pain catastrophizing and, through it, to prescription opioid misuse. We also addressed the potential mechanisms which could link pain catastrophizing and prescription opioid misuse: heightened depression, anxiety, and pain intensity. Our results showed that pain catastrophizing partially mediated the relationship between impulsivity and prescription opioid misuse, thus supporting H1. As predicted, depression and anxiety partially serially mediated the relationship between pain catastrophizing and prescription opioid misuse; however, pain intensity did not mediate this relationship, and thus, H2 was only partially supported. In summary, the results of the current study showed associations between increased impulsivity and increased pain catastrophizing, between increased pain catastrophizing and higher depression and anxiety, and between higher depression and anxiety and greater prescription opioid misuse.

The results highlight the key role of impulsivity in relation to prescription opioid misuse. Impulsivity was indirectly associated with prescription opioid misuse through pain catastrophizing, anxiety, and depression, and also had a significant direct effect. The few studies on the role of impulsivity in prescription opioid misuse have shown that only a reduced set of dimensions are relevant. However, the results are contradictory. Marino et al. [10] found that of the three BIS-11 [11] subscales, only attentional impulsivity—the capacity to control attention—was significantly associated with risk of opioid analgesic misuse in patients with chronic pain, whereas Vest et al. [8] found that of the dimensions of impulsivity, only urgency—defined as the tendency to react impulsively in response to negative emotional states—was associated with the risk of future and current misuse of prescription opioids, as well as symptoms of current opioid use disorder. In the current study, our initial objective was to investigate the different roles of each of the dimensions of impulsivity in relation to prescription opioid misuse. Unfortunately, this goal could not be fulfilled because we found that the BIS-11 subscales had low reliability. We are aware that this is a relevant limitation of the study given that the identity of impulsivity as a unitary construct has been challenged [22]. Future research should test the relationships postulated in the current study using the specific dimensions of the construct of impulsivity.

The results showed a direct association between impulsivity and prescription opioid misuse and an indirect association through increased pain catastrophizing. A significant positive correlation was found between impulsivity and pain catastrophizing. Only a few studies have specifically investigated the relationship between impulsivity and pain catastrophizing. Harmanci and Horasanli [40], in women with primary dysmenorrhea, and Harmanci and Kul [41], in people with chronic migraine, found that both constructs were positively and significantly associated. There is consistent evidence suggesting that impulsivity and pain catastrophizing could be related through their common attentional components: one of the main dimensions of impulsivity refers to the capacity to focus selectively on specific stimuli and avoid interference [22]. Pain-related catastrophic thoughts are characterized by their ruminative nature, and there is strong evidence that rumination—as the disengagement hypothesis suggests [42]—is the reflection of attentional control deficits. In fact, there is also strong evidence of an association between pain catastrophizing and the inability to disengage from pain-related stimuli and information [24].

The current study showed an association between impulsivity and prescription opioid misuse through heightened anxiety and depression. De Carlo et al. [43] found a strong positive association between impulsivity and anxiety in impulse control disorders. In this regard, research has focused in particular on emotion-related impulsivity, that is, the tendency to engage in rash and regrettable speech and behavior and unconstrained cognition when faced with high negative emotions (negative urgency) and high positive emotions (positive urgency) [23]. Research has shown an association between both high negative and positive urgency and anxiety [44] and depression [45, 46], and addiction problems through anxiety [47] and depression [48]. In this regard, as mentioned, Vest et al. [8] found a significant association between the urgency dimension of impulsivity and risk for future misuse and current misuse of prescription opioids as well as symptoms of current opioid use disorder.

Our results are consistent with previous studies that have found high comorbidity between anxiety, depression, and opioid misuse (e.g., [49]). An integrative model suggests a bi-directional relationship between opioid misuse and anxiety/depression [49]. It also posits that negative reinforcement perpetuates addiction through a learned association between relief from aversive internal states (such as negative affect and pain) and opioid intake [49].

Apart from the direct association between anxiety and depression and prescription opioid misuse and their mediation of the relationship between impulsivity and misuse, we found that they significantly mediated the relationship between pain catastrophizing—in itself a mediator—and prescription opioid misuse, which is in line with the results of previous studies [18]. This finding highlights the role of pain catastrophizing as an ineffective emotion regulation strategy which results in increased emotional distress [15]. It is also in line with psychopathological models which highlight the role of rumination as a transdiagnostic factor in anxiety and depression disorders [50]. Interestingly, our results are similar to those of a longitudinal study that found that both rumination and impulsivity (urgency) predicted unique variance in future depression [46]. Finally, as previous research has also shown [13], we found an association between pain intensity and pain catastrophizing; however, contrary to expectations, pain intensity did not mediate the relationship between pain catastrophizing and prescription opioid misuse. Although pain control seems to be a powerful self-reported motive for opioid misuse distinct from negative affect coping motives [51], we found that the association between prescription opioid misuse and anxiety and depression was stronger than its association with pain intensity. Although individuals with chronic pain may consider that their opioid consumption is dictated by their pain levels, we suggest that it is in fact determined by the emotional distress associated with the entire chronic pain experience. Future research may shed light on this dichotomy.

This study has several limitations. It has a cross-sectional design, and therefore causal conclusions cannot be drawn regarding the associations between the study variables. Additionally, it relies exclusively on self-report instruments. Future research could incorporate more objective measures, such as blood and urine tests for opioid use. Another limitation of this study is that we only considered the total pain catastrophizing score; future research should examine the potential roles of the dimensions of this construct. Finally, the study sample included a higher proportion of women than men, which may have introduced bias. Future research using a more sex-balanced sample would enable testing the model across both groups to determine whether the mechanisms in the model operate differently in women and men.

Given the association between impulsivity and prescription opioid misuse through emotional distress and pain catastrophizing, two targets for clinical intervention would appear to be effective. On the one hand, emotion-related impulsivity could be addressed by brief interventions that teach individuals to recognize emotions, engage in self-calming techniques in response to emotional states, and pre-plan coping strategies to manage high emotional states [52]. On the other hand, cognitive control training interventions have demonstrated effectiveness in treating deficits in cognitive control and reducing rumination, even over the long term (e.g., [42]).

### 4.1. Conclusions

Impulsivity plays a key role in relation to prescription opioid misuse. Associations were found between increased impulsivity and increased pain catastrophizing, between increased pain catastrophizing and higher depression and anxiety, and between higher depression and anxiety and greater prescription opioid misuse. An indirect association was also found between impulsivity and prescription opioid misuse through pain catastrophizing, anxiety, and depression.

## Figures and Tables

**Table 1:** Demographic and clinical variables (*N* = 366).

**Demographic variables**	**%**

Sex	
Female	65
Male	35
Marital status	
Single	12
Married	67
Divorced/separated	14
Widowed	7
Education	
Reading and writing	12
Primary school	30
High school	43
University education	15
Work status	
Homemaker	5
Employed	24
Student	1
Unemployed	19
Retired	51

	*M* (SD)

Age (years)	58.2 (12.03)

**Clinical variables**	**%**

Diagnosis	
Primary chronic pain	34
Secondary chronic pain	
Postsurgical/Post-traumatic	14
Neuropathic pain	7
Orofacial and headache	1
Musculoskeletal	44
Adjuvant medications for treating pain	
Benzodiazepines	62
Antidepressants	65
Antiepileptic drugs	55
Hypnotics	6

	*M* (SD)

Time in pain (months)	203.1 (171.2)
Daily MME/day	56.8 (77.9)
Time in treatment with opioids (months)	53.5 (55.4)

Abbreviation: MME = morphine milligram equivalents.

**Table 2:** Means, standard deviations, and Pearson correlation coefficients (*N* = 366).

Variables	*M* (SD)	1	2	3	4	5	6
1. Impulsivity	42.95 (14.98)	1	0.23^∗∗^	0.48^∗∗^	0.31^∗∗^	0.10^∗^	0.35^∗∗^
2. Pain catastrophizing	36.30 (10.39)		1	0.53^∗∗^	0.48^∗∗^	0.30^∗∗^	0.42^∗∗^
3. Anxiety	18.55 (5.85)			1	0.63^∗∗^	0.29^∗∗^	0.49^∗∗^
4. Depression	15.95 (5.81)				1	0.21^∗∗^	0.48^∗∗^
5. Pain intensity	6.61 (1.55)					1	0.17^∗^
6. Opioid misuse	13.20 (8.86)						1

*Note: M* = mean.

Abbreviation: SD = standard deviation.

⁣^∗^*p* < 0.01.

⁣^∗∗^*p* < 0.001.

**Table 3:** Results of serial mediational analyses.

**Pain catastrophizing (M1) and depression (M2) as mediators between impulsivity (X) and prescription opioid misuse (Y)**					
Direct^1^					
	**B**	**SE**	***p***	**LLCI**	**ULCI**

IMPUL ⟶ CATAS (*a*_1_)	0.16	0.04	0.000	0.086	0.226
IMPUL ⟶ DEPRE (*a*_2_)	0.08	0.02	0.000	0.048	0.118
CATAS ⟶ DEPRE (*d*)	0.24	0.03	0.000	0.189	0.290
CATAS ⟶ MISUSE (*b*_1_)	0.19	0.04	0.000	0.104	0.271
DEPRE ⟶ MISUSE (*b*_2_)	0.48	0.08	0.000	0.324	0.630
IMPUL ⟶ MISUSE (*c*′)	0.12	0.03	0.000	0.069	0.176

Indirect^2^					
	**B**		**SE**	**BooLLCI**	**BooULCI**

Total	0.15		0.03	0.095	0.209
IMPUL ⟶ CATAS ⟶ MISUSE	0.05		0.02	0.025	0.086
IMPUL ⟶ CATAS ⟶ DEPRE ⟶ MISUSE	0.03		0.01	0.015	0.052
IMPUL ⟶ DEPRE ⟶ MISUSE	0.07		0.02	0.034	0.110

**Pain catastrophizing (M1) and anxiety (M2) as mediators between impulsivity (X) and prescription opioid misuse (Y)**					
Direct^1^					
	**B**	**SE**	***p***	**LLCI**	**ULCI**

IMPUL ⟶ CATAS (*a*_1_)	0.16	0.04	0.000	0.086	0.226
IMPUL ⟶ ANX (*a*_2_)	0.15	0.02	0.000	0.115	0.178
CATAS ⟶ ANX (d)	0.25	0.02	0.000	0.206	0.297
CATAS ⟶ MISUSE (*b*_1_)	0.19	0.05	0.000	0.105	0.280
ANX ⟶ MISUSE (*b*_2_)	0.43	0.09	0.000	0.263	0.607
IMPUL ⟶ MISUSE (c')	0.10	0.03	0.001	0.040	0.157

Indirect^2^					
	**B**		**SE**	**BooLLCI**	**BooULCI**

Total	0.19		0.03	0.127	0.253
IMPUL ⟶ CATAS ⟶ MISUSE	0.05		0.02	0.025	0.088
IMPUL ⟶ CATAS ⟶ ANX ⟶ MISUSE	0.03		0.01	0.013	0.053
IMPUL ⟶ ANX ⟶ MISUSE	0.11		0.02	0.065	0.159

**Pain catastrophizing (M1) and pain intensity (M2) as mediators between impulsivity (X) and prescription opioid misuse (Y)**					
Direct^1^					
	**B**	**SE**	***p***	**LLCI**	**ULCI**

IMPUL ⟶ CATAS (*a*_1_)	0.16	0.04	0.000	0.086	0.226
IMPUL ⟶ PAIN (*a*_2_)	0.00	0.01	0.457	−0.007	0.014
CATAS ⟶ PAIN (*d*)	0.04	0.01	0.000	0.029	0.059
CATAS ⟶ MISUSE (*b*_1_)	0.29	0.04	0.000	0.210	0.374
PAIN ⟶ MISUSE (*b*_2_)	0.22	0.27	0.424	−0.320	0.760
IMPUL ⟶ MISUSE (c')	0.16	0.03	0.000	0.106	0.216

Indirect^2^					
	**B**		**SE**	**BooLLCI**	**BooULCI**

Total	0.30		0.10	0.147	0.561
IMPUL ⟶ CATAS ⟶ MISUSE	0.28		0.10	0.137	0.535
IMPUL ⟶ CATAS ⟶ PAIN ⟶ MISUSE	0.01		0.01	−0.012	0.039
IMPUL ⟶ PAIN ⟶ MISUSE	0.01		0.01	−0.009	0.056

*Note:* IMPUL: impulsivity; CATAS: pain catastrophizing; DEPRE: depression; MISUSE: prescription opioid misuse; BooLLCP: bootstrap lower limit confidence interval; BooULCP: bootstrap upper limit confidence interval.

Abbreviations: LLCI = lower limit confidence interval, SE = standard error, ULCI = upper limit confidence interval.

^1^The reported effects are standardized coefficients.

^2^The reported effects are completely standardized coefficients.

## Data Availability

Templates available at Mendeley Data: https://doi.org/10.17632/pcm7c3z6vc.1.
